# Characterizing Topics in Social Media Using Dynamics of Conversation †

**DOI:** 10.3390/e23121642

**Published:** 2021-12-07

**Authors:** James Flamino, Bowen Gong, Frederick Buchanan, Boleslaw K. Szymanski

**Affiliations:** 1 Department of Computer Science, Rensselaer Polytechnic Institute, Troy, NY 12180, USA; flamij@rpi.edu (J.F.); boweng@gmail.com (B.G.); buchaf@rpi.edu (F.B.); 2 Department of Physics, Applied Physics, and Astronomy, Rensselaer Polytechnic Institute, Troy, NY 12180, USA; 3 Społeczna Akademia Nauk, Henryka Sienkiewicza 9, 90-113 Łódź, Poland

**Keywords:** information dynamics, topic modeling, semantic analysis, network entropy

## Abstract

Online social media provides massive open-ended platforms for users of a wide variety of backgrounds, interests, and beliefs to interact and debate, facilitating countless discussions across a myriad of subjects. With numerous unique voices being lent to the ever-growing information stream, it is essential to consider how the types of conversations that result from a social media post represent the post itself. We hypothesize that the biases and predispositions of users cause them to react to different topics in different ways not necessarily entirely intended by the sender. In this paper, we introduce a set of unique features that capture patterns of discourse, allowing us to empirically explore the relationship between a topic and the conversations it induces. Utilizing “microscopic” trends to describe “macroscopic” phenomena, we set a paradigm for analyzing information dissemination through the user reactions that arise from a topic, eliminating the need to analyze the involved text of the discussions. Using a Reddit dataset, we find that our features not only enable classifiers to accurately distinguish between content genre, but also can identify more subtle semantic differences in content under a single topic as well as isolating outliers whose subject matter is substantially different from the norm.

## 1. Introduction

Consider this question: if a person were to go to a movie theater and see a random movie without knowing the title or anything else about it beforehand, how could this person guess the movie’s genre while watching it? One way would be to examine the title and characters’ dialogue. Another way would be to analyze the visuals and special effects. However, a more atypical approach might be to turn around and look to the audience for the answer. If we specifically do not want to use dialogue or visuals, we can use the audience’s reactions. For example, if the audience is laughing frequently, it is likely to be a comedy, but if they are mostly crying out in fear, the film is probably a horror movie. While there are exceptions, the process described is reliable enough, given that an audience’s emotional reaction is inherently tied to the nature of a movie’s genre. After all, the director of a horror film needs the audience to react in fear; otherwise, their film will most likely fail.

While this concept as applied through our example is unconventional, it illustrates a fascinating concept in the realm of social media. Hence, we conjecture that just as these emotional reactions from an audience fundamentally describe the associated movie, topics in online social media have their own set of unique “user reactions” that can be extracted and used to characterize the unique properties of the discussed subject matter without any help from text analysis. Specifically, we hypothesize that if a group of users are biased in a certain way, they will react differently in more ways than what text analysis will reveal. An example supporting this hypothesis can be found in [1], where the authors showed that, within Twitter, users have stable and consistent reactions associated with a given topic, called a “Social Genotype Model”. However, this stable representation of bias is only sparsely applied by the authors, being primarily used for the prediction of topic-specific influencers and the high-level analysis of Twitter’s follower network. Moreover, while extracting user reactions has been seen as an essential tool for social media analysis for a while [2,3,4,5], most implementations tend to be limited to analyzing verbal responses using natural language processing (NLP) and the quantification of baseline interest. Furthermore, whatever structural response analysis does exist [6] is often used to study individual and aggregate users, rather than examine the characteristics of topics.

Previous NLP work has established a paradigm for understanding human interactions and the content they generate [7], but in this paper, we will explore the relationship between the dynamics of discourse, conversation, and topic within social media without the use of direct textual analyses. Instead of using NLP as our primary mode of analysis, we opt for an alternative, novel approach of using text-agnostic, reaction-based measures that provide significant insight into the understanding of topics in online social media. We show that this approach not only allows us to identify topics and differentiate topical divisions, but also capture more subtle semantic differences within individual topics. Provided the mathematical quantification of discourse dynamics, we can use established outlier detection tools, such as isolation forests [8], to identify content that is, according to our measures, substantially different from the content of the norm. We subsequently investigate the content of these identified outliers and discuss their various aspects that contribute to their differences from the norm.

In this paper, we begin our exploration of our novel approach by introducing the dataset we use in Section 2.1. We then describe the response features we chose to use, their mathematical quantification, and the methods we use to employ them in Section 2.2. Next, we explain the tests we use to validate and explore the capabilities of the response features in Section 2.4, Section 2.5 and Section 2.6. We present and analyze the results of these tests in Section 3, and conclude our work with a overarching discussion of the paper in Section 4.

We note that this paper is an extension of our conference paper: Flamino, J.; Szymanski, B.K. A Reaction-Based Approach to Information Cascade Analysis. 28th Int. Conf. Comput. Commun. Netw. IEEE, 2019, pp. 1–9 [9]. We have significantly extended this initial work by adding substantial new analyses and results and revising some of our original content. Specifically, we improve our feature clustering analysis by introducing a PageRank method for extracting keywords. This allows us to add a word cloud illustration analysis that corroborates the semantic separation of the clusters. We also introduce an entirely new correlative analysis between top 10 keyword lists of feature cluster pairs and the distances between their centroids. Furthermore, we perform a new latent Dirichlet allocation (LDA) comparative analysis, comparing LDA topic distributions against the response feature clusters. Finally, we expand upon the outlier detection process by introducing a different outlier detection algorithm (isolation forest) and by adding an entirely new outlier example with improved descriptions of the submissions we were analyzing.

## 2. Materials and Methods

### 2.1. Datasets

Given our focus on the dynamics of discourse, we need to analyze a dataset from an online social media platform that encourages in-depth discussions of a wide variety of topics and subjects. The format that we found to best fit this description was the group of online platforms called *forums*. A forum is a network of registered users in which any user can freely submit posts about certain topics (generally under some related category). This post triggers responses to the posted material. In turn, these responses trigger more responses, resulting in a cascade of information exchanged between unique users. A majority of these cascades are short-lived, as they are quickly superseded by more recent topics. Yet, the conversations that do occur due to that post follow the theme established by the source post, characterizing a majority of the interactions that occur within that cascade.

One of the most well-known forum-like social media platforms is Reddit. In Reddit, the posts are clustered by Subreddit, which generally encompasses a defining theme (e.g., the Subreddit r/politics is comprised of discussions about U.S. politics). Within a Subreddit, a user can create a submission pertaining to the Subreddit’s genre. The submission then becomes available to all other users. Users can vote on the quality of the submission and start discussions in the comment section of the submission. The more provocative the subject of a submission, the greater the response, ultimately increasing the activity of the submission and the vote count, which inevitably increases the exposure of the submission, evoking additional responses.

We use the data provided from [10], which offers a Reddit dataset that covers 5692 Subreddits, 88 million submissions, and 887.5 million comments collected from 2006 to 2014. The comments in this dataset are formatted as a comment tree extension, accentuating the natural branching conversations that sprout from the source submission. We reformat this structure into event sequence style, where the identifiers id, root_id, and parent_id reveal the event’s position in a cascade. To clarify, the root_id indicates the id of the source submission, while the parent_id indicates the id of the event that the posting user is responding directly to. We also supplement the 88 million submissions with their respective titles, text bodies, and scraped headlines from any linked URLs, using Reddit’s official API.

### 2.2. Characterizing the Dynamics of Conversation and Discourse

Our objective is to use the dynamics of conversation and discourse to characterize and understand the overarching topic. With respect to Reddit, this means we use the comment tree to identify the theme of the submission without any text mining from the comment tree and even from the submission itself. Instead, we capture attributes relating to the structure and evolution of the comment trees. Some past works have established groundwork on harnessing such response structures within social media like Twitter [1] and Reddit [11]. As mentioned in the introduction, ref. [1] demonstrates in particular consistent topic-dependent user behavior, which can be used to reinforce our hypothesis that users will oftentimes respond differently as a whole depending on the subject matter.

#### 2.2.1. Definitions

Given Reddit’s emphasis on forum-like interactions, the emergent online social media network G(M,L) is not formed in a typical fashion, where edges connect unique users m∈M like $l=(m_{i},m_{j}),\;l\in L$. While users can “follow” other users in Reddit, the more apparent link to content is through the subscription to Subreddits. Once subscribed, a user becomes part of a collective of fellow subscribers that are all updated when any other subscribed users post a submission to that Subreddit. This leads us to Observation 1.

**Observation** **1.** *Reddit can be considered a set of Subreddit tags S. Each Subreddit has a set of associated users M. All users m in set M are connected with an edge representing information flow. This system forms an isolated, undirected complete graph.*

Naturally, the network of Reddit becomes more complicated once you consider that users subscribe to multiple Subreddits and follow other users, but when considering the graph topology within the scope of a single Subreddit, the observation holds. Given this approach, we can intuitively state that submissions within a Subreddit are also generally detached from each other. The graphical topologies of these submissions are fundamentally different since responses to a source node stack on top of each other. This leads us to make Observation 2.

**Observation** **2.** *Each submission that occurs within a Subreddit is an independent information cascade that represents a set of users* r={m}, r⊆M *interacting for some period of time, with the resultant event sequence following the graph topology of a directed tree network.*

**Figure 1:**
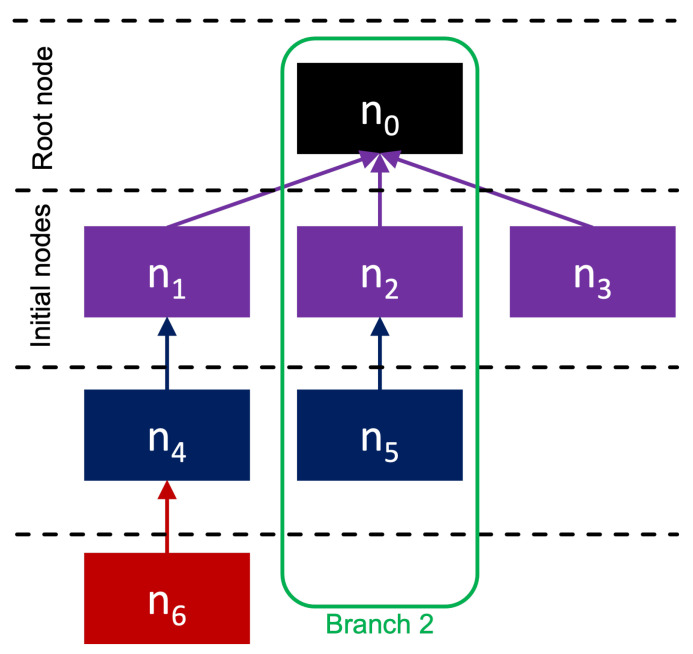
Representation of a directed tree network *T*.

We denote as T(N,L) the event sequence generated by the users of some submission *r*, where each n∈N represents a message somewhere in the event sequence triggered by said submission. A connecting edge is defined as $l=(n_{i},n_{j}),\;l\in L$, where $n_{j}$ is the responder’s message, and $n_{i}$ is the respondee’s message. It is important to note that not every message *n* has a uniquely associated user *m*. While each node *n* is unique, a user has free reign to generate as many nodes as they please, resulting in user degeneracy in *T*. We illustrate this terminology and the architecture of *T* in Figure 1. As seen in Figure 1, we refer to $n_{0}$ as the *root node*, and all nodes that directly link to the root node are referred to as *initial nodes*. All other nodes are simply referred to as *response nodes*. We can also extract a subset of directly correlated messages called branches. Branches are represented by $B=\{B_{1},B_{2},\ldots ,B_{b}\}$ where *b* is equal to the total number of initial nodes. We can use some $i^{\mathrm{th}}$ branch $B_{i}$ where $B_{i}\subseteq N$ to evaluate the response cascade that is triggered by the $i^{\mathrm{th}}$ initial node. We note that for every branch, we include the root node as an element of the set. This is because for all intents and purposes, the root node is the submission’s content itself; thus, we must include this node to ensure our features capture that initial reaction from users that directly respond to the submission. Table 1 summarizes the mentioned symbols. Now that we have established our definitions, we can begin constructing our response features from the empirical data.

#### 2.2.2. Feature Design

To characterize the dynamics of discourse and conversation in Reddit, we split our features into two sets: individual features and aggregate features. For the individual features, the real values must describe the nature of user interactions within a single branch $B_{i}\in B$. To generate these values, we design features that capture innate behavior exhibited in both scale and time without recursively analyzing each involved user or message. We shall refer to these as our *individual features*.

1. *Depth*: The path length between the root node and the farthest response node in a branch. We use Dijkstra’s algorithm (DA) to measure this.
  $$\mathrm{DEP}\left(B_{i}\right)=\max_{n\in B_{i},n\neq n_{0}}\left(\mathrm{DA}\left(n_{0},n\right)\right)$$
2. *Magnitude*: The maximum in-degree centrality in a branch. Given the adjacency matrix *A* for *T*, then
  $$\mathrm{MAG}\left(B_{i}\right)=\max_{n\in B_{i}}\left(\sum_{k}a_{k,n}\right),a_{k,n}\in A$$
3. *Engagement*: The total number of users involved in a branch where $n_{m}$ is the set of messages generated by user *m*.
  $$\mathrm{ENG}\left(B_{i}\right)=\left|\{m\;|\;\right|n_{m}\cap B_{i}\left|>0\}\right|$$
4. *Longevity*: The time *t* that expired between the creation of an initial node and the latest response node.
  $$\mathrm{LNG}\left(B_{i}\right)=\max_{n\in B_{i}}\left(t\left(n\right)\right)-\min_{n\in B_{i},n\neq n_{0}}\left(t\left(n\right)\right)$$

In addition to these four features, we introduce two additional holistic features, raising the number of features to six. We use these holistic features to evaluate a submission’s aggregate discourse dynamics within *T*, independently of *B*. These methods are extracted from [12] in which the authors evaluate human communication in temporal networks using entropy and introduce entropy-based measures for human communication. In particular, we chose to consider both the first and second order entropy measures that were introduced. These two methods produce distinct numerical representations that portray aggregate comment interactions succinctly. Thus we refer to these measures as our *aggregate features*.

5. *First order entropy*: The probability $p_{1}\left(m\right)$ of some user *m* within *r* generating a message *n* for *T*.
  $$E_{1}=-\sum_{m\in r}p_{1}\left(m\right)\ln\left(p_{1}\left(m\right)\right)$$
6. *Second order entropy*: The probability $p_{2}\left(l_{ij}\right)$ of a unique edge $l_{ij}=\left(n_{i},n_{j}\right)$ being formed between two messages by two specific users $m_{i}$ and $m_{j}$ within *T*.
  $$E_{2}=-\sum_{l_{ij}\in L}p_{2}\left(l_{ij}\right)\ln\left(p_{2}\left(l_{ij}\right)\right)$$

For the remainder of the paper, we use these features to characterize the response dynamics in Reddit. Employing the individual measures for some response *r* yields a matrix $M_{r}$ of dimensions b×z where b=|B| and z=4 for the 4 individual features defined above.

$$M_{r}^{T}=\left[\begin{array}{cccc} \mathrm{DEP}(B_{1}) & \mathrm{DEP}(B_{2}) & \cdots & \mathrm{DEP}(B_{b})\\ \mathrm{MAG}(B_{1}) & \mathrm{MAG}(B_{2}) & \cdots & \mathrm{MAG}(B_{b})\\ \mathrm{ENG}(B_{1}) & \mathrm{ENG}(B_{2}) & \cdots & \mathrm{ENG}(B_{b})\\ \mathrm{LNG}(B_{1}) & \mathrm{LNG}(B_{2}) & \cdots & \mathrm{LNG}(B_{b}) \end{array}\right]$$

Matrix $M_{r}$ describes our individual features for all branches of *r*. Combined with our aggregate features, we have a compact yet detailed portrayal of the dynamics of a submission’s entire discourse.

### 2.3. Validating Response Features through Genre Classification

With the methods for discourse characterization defined, a necessary next step is to validate the actual effectiveness of the selected features in the scope of our dataset. In particular, we need to assess how accurately these features represent the topics of the Reddit submissions they are quantifying. We can do this through a Subreddit classification process: given a few distinct Subreddits acting as labels, we use the extracted response features from a subset of submissions to train a classifier to distinguish between said labels. In essence, we investigate if our features’ characterizations of some set of submissions are enough to train a classifier to differentiate between these submissions’ genres. We run this test on the Subreddits r/politics, r/gaming, r/soccer, and r/atheism, each representing an independent genre within Reddit. We curated a set of 1000 submissions from each Subreddit, only selecting submissions whose total comment counts range from 1000–3000 comments (see the Supplementary Materials for an explanation as to why we use this specific comment range for submission filtering). Then for each submission, we calculate $M_{r}$ and the aggregate features. We condense $M_{r}$ by finding the maximum depth (the height of the tree), average breadth, average engagement, and average longevity for each submission. We map the submission’s measures into $R^{6}$ feature space by taking these values and appending to them the aggregate features. We can represent this process as the equation

$$\begin{array}{ccc} f & = & <\max_{B}\left(DEP\right),\underset{B}{\mathrm{avg}}\left(MAG\right),\underset{B}{\mathrm{avg}}\left(ENG\right),\underset{B}{\mathrm{avg}}\left(LNG\right),E_{1},E_{2}> \end{array}$$

where vector *f* is our feature vector. Pairing *f* with its associated Subreddit label *S*, we then split the assembled dataset into 70%/30% training/testing subsets and train a support vector machine classifier (SVM) [13]. For each element in the test subset, the SVM attempts to correctly label *S*, given *f* for *r*. A positive match results in a score of 1 for *r*, and a negative match yields a 0.

### 2.4. Feature Exploration through Clustering

Although classification is a fairly robust way of testing the effectiveness of a set of feature labels, there are many other ways to evaluate them. In particular, these features are meant to represent the patterns of user response within a submission. Knowing this, another effective form of testing is to use the features to attempt to find underlying patterns that differentiate clusters of submissions within a single genre. If this is possible, it would indicate that the response features themselves are rich and informative enough to extract previously undetected trends and sub-divisions of content.

To perform the clustering analysis, we evaluate a set of 1000 submissions under a single *S* whose total comment counts range from 1000–3000 comments. Here, we use K-means for our clustering algorithm for its simplicity, speed of convergence, and centroid-based hard assignments (which we use further in a complementary analysis, described in the following paragraph). Using K-means paired with the silhouette score [14] to determine the *K*, we can find distinct clusters among the submissions of the same label. We define these clusters as $C=\{C_{1},C_{2},\ldots ,C_{K}\}$, where *K* is the cluster number given the highest average silhouette score. Provided the clusters, we need to validate that the clusters into which the content is separated are divided in a way that can be interpreted by humans. To do this, we implement a NLP method to accurately summarize the content that is being clustered. For a cluster $C_{k}$, we aggregate the titles and text bodies (but not comment text) of all submissions belonging to that cluster into a single document. We then extract all nouns (and proper nouns) and project them into a network, where each node represents a unique keyword. Note that a keyword can either be a word or a sequence of words that occur together consistently in the considered text. The edge between two nodes represents keyword co-occurrence, where the weight of that edge is equal to the total number of times that pair of keywords has co-occurred in any of the sentences in the joined text. We then run PageRank [15] on the resultant network in order to identify the keywords in our text that have a significant presence. We extract all non-trivial keywords, as identified by PageRank, and count the occurrence frequency of these keywords as they appear within said clusters. If the extracted keywords associated with each cluster have minimal (or no) overlap, this indicates that the response features used to identify the detected clusters have done an effective job in grouping topic using our text-agnostic quantification of discourse dynamics.

With K-means and our keywords, we are also able to explore if the separation in content correlates with the distances between clusters. Given a set of clusters where K>2, we extract from each a list of the top 10 keywords as ranked in descending order by PageRank. We then find the Euclidean distance between each pair of cluster centroids. To quantify the content difference between each pair, we use ranked bias overlap (RBO) [16], a list comparison measure, on the two top 10 keyword lists associated with the two submissions in consideration. With RBO, the lower the score, the greater the difference in the keyword lists, with emphasis on the higher-ranking keywords. When RBO is at 0, there is no overlap in the top 10 keyword lists extracted from the pools of content of the two submission clusters in question. By plotting the RBO scores against the Euclidean distances of each pair of cluster centroids, we are then able to more directly observe a quantifiable relationship between our response features and the topics they cluster together. If our hypothesis holds, the farther apart any two clusters are, the less overlap in terms of content there should be, as the comment structures should be increasingly different.

### 2.5. Comparing Response Features to Latent Dirichlet Allocation

This system of submission clustering could be considered a form of non-semantic topic modeling, as the process is designed to identify distributions of stable topics exclusively through discourse dynamics, with each cluster representing a set of keywords (or submissions) that in itself represents a semantic grouping (or topic). As such, it makes sense to compute a correlative analysis between our response feature clustering approach and the topical assignments made through the traditional NLP topic modeling methodology. Specifically, we explore the topic distributions of the submissions we cluster through response features using a latent Dirichlet allocation model (LDA) [17].

For a clustering sample used in our response feature analysis, we pool the titles and text bodies of the submissions in the sample. Then for the LDA model, we set the number of topics to the value of *K* previously assigned to the sample. The model produces *K* number of topics, each associated with a combination of keywords. Treating the sample’s submissions as independent documents, the LDA model assigns each submission a distribution of topics, with each topic being weighted by the contribution that that topic makes to that submissions. As K-means uses hard assignments, for comparison, we label each submission with their dominant topic. Then, we project the submissions into 2D space again using the response features. We assign the submissions their K-means cluster labels as well as their LDA topic labels in order to see if there is similar groupings. A similar grouping would indicate there is a correlation between the topics that the LDA model finds and the groupings the text-agnostic response features have extracted.

### 2.6. Outlier Detection

A natural extension of analyzing the topical differences of clusters of submissions is to implement outlier detection. If the response features can accurately separate content through discourse dynamics, an outlier, as characterized by those response features, would naturally indicate that the topic of the outlier submission is quite different from those that were grouped together. Like the previous section, to investigate the importance of outliers as detected through response features, we evaluate a set of 1000 submissions (within the 1000–3000 comments range) under a single *S*. Provided with the response features for all submissions, we use the isolation forest outlier detection algorithm to identify what submissions are substantially different from the others and investigate the content of those submissions. An isolation forest is a collection of decision trees that isolate submission outliers by randomly choosing a feature from our response features, then randomly selecting a split value that is between the available minimum and maximum values of this feature. Given enough data to help establish a trend of the norm, this random partitioning of features should generate shorter paths in the trees for outliers, which distinguishes these anomalous data points from the rest. The isolation forest method operates on the principle that outliers are observations that are few and different from the norm, which allows us to use the resultant shorter paths to act as our identifiers. For each point, the isolation forest outputs an anomaly score that has the range of [−1,1]. The more negative the score, the more likely the point in question is an anomaly, or outlier. The more positive, the more likely the data are part of the norm.

## 3. Results

### 3.1. Genre Classification

For our SVM genre classification process, Table 2 shows the classifier scores between several pairs of label sets. See Supplementary Materials Figure S1 for corresponding confusion matrices of these classifications. The average score is 0.85, indicating that the patterns of conversation are distinctly different between Subreddits due to their themes. In general, people will not talk about games in the same ways they will talk about religion. This innate difference in conversation style contributes enough to the overall structure so that an SVM can accurately classify a submission using only the six response features. We observe that the scores in Table 2 can also be seen as a high-level quantification of the similarities and differences between themes. For example, while the classification score between r/politics and r/soccer is 0.96, the score between r/politics and r/atheism is 0.76. In agreement with our intuition, even though intense debates are certainly known to the world of soccer, the depth and intensity of debates that sprout up from every aspect of politics provide a clear dichotomy in comment structure. However, the atheism Subreddit (and the religious genre in general) also provides an environment for continuous in-depth debate due to its religious connotations, which might be consistent enough to be more similar to politics. For robustness, we also use this SVM process to explore the differences between response features extracted from submissions from the time range of 2006 to 2010 and response features from the time range of 2011 to 2014. See Supplementary Materials for details and results.

### 3.2. Clustering Analysis

**Figure 2:**
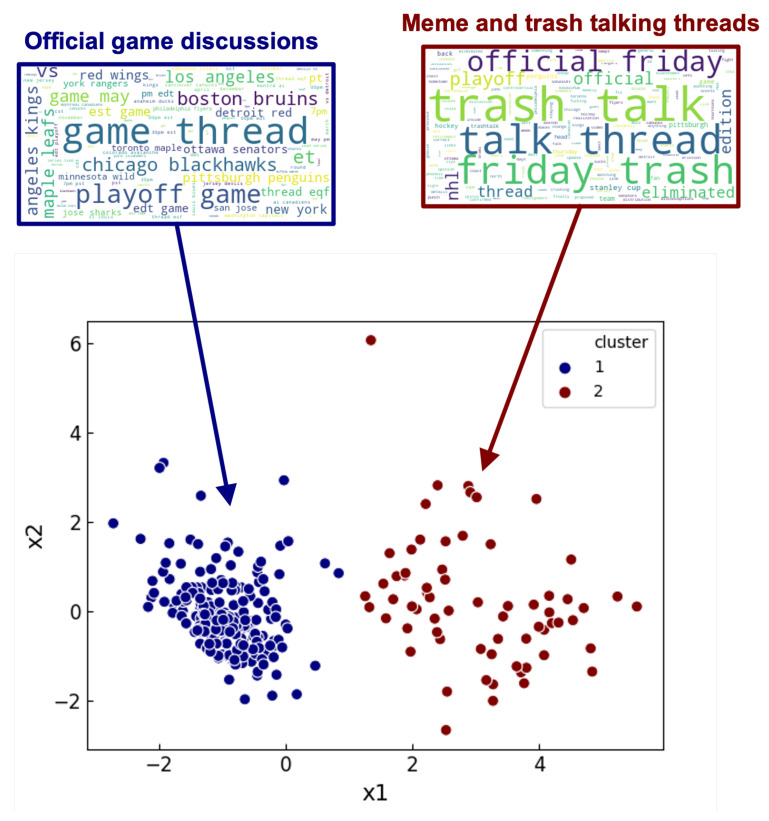
PCA of response features for 1000 submissions from r/hockey, clustered by K-means (K = 2). Word clouds of keywords extracted from the text of the submissions contained in each cluster are shown above together, with the size of each keyword corresponding to the magnitude of their respective PageRank score.

For our analysis of clustering under a genre, we consider r/hockey as our first example. We use K-means on 1000 submissions within the 1000–3000 comment range for K=2. After clustering, we use our keyword extraction process to extract all prevalent keywords from the pool of submission texts in $C_{1}$ and $C_{2}$. For visualization purposes, we implement principle component analysis (PCA) to reduce the $R^{6}$ dimensionality to $R^{2}$. This reduces each submission’s set of response features to a two-dimensional point that can be projected onto a 2D plot, allowing us to view the differences in response features easily. We plot our 1000 submissions, color coded by their associated cluster, in Figure 2. To complement the plot, we also illustrate the extracted keywords in word clouds linked to the clusters, where the size of the words in the clouds corresponds to the magnitude of their PageRank score.

As seen in Figure 2, there are two apparent clusters, delineating a difference in comment structure within the Subreddit itself. Using the word clouds complemented with a cursory textual analysis of the involved submissions, we see that cluster 1 contains submissions pertaining to official “Game Thread” discussions, while cluster 2 is primarily related to trash talking and memes. We further reinforce our word cloud observation by also counting the frequency of occurrence of some of the top keywords in the text pools of both clusters, shown in Table 3.

From Table 3, we can see that the keyword occurrence is asymmetric, with little overlap between the highest occurring keywords of the first and second cluster. When paired with our other observations, these results indicate that the two r/hockey clusters, while under the same genre, are still semantically different, involving different types of discussions therein. Intuitively, we know there is a difference between official game discussions and trash talking in the ways in which the corresponding conversations are carried out, and these differences are captured by our response features through K-means.

**Figure 3:**
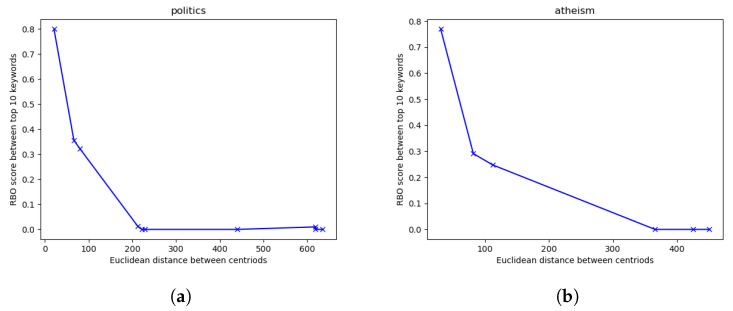
RBO score of top 10 keyword lists versus Euclidean distance of response feature clusters for (**a**) r/politics where K=5 and (**b**) r/atheism where K=3. RBO’s weight parameter *p* is set to 0.98.

As the two sets of keywords for our two clusters in r/hockey are clearly separate in both content and in Euclidean space, the next step is to investigate the relationship between cluster distance and content differences. To do this, we use 1000 submissions from r/politics, finding their response features and then clustering them through K-means where K=5. The result for this is shown in Figure 3a. For robustness, we repeat the above process for r/atheism as well, where K=3. These results are shown in Figure 3b.

### 3.3. LDA Analysis

We trained an LDA model on the r/science and r/news Subreddits, selecting 1000 submissions with a comment range of 1000–3000 within these genres. Extracting our response features from these samples, we find that the best K for the K-means process is K=3 for r/science while K=2 for r/news. Using this for the number of topics in our LDA model, we found for r/science that the average dominant topic for each submission had a 90.7%±8.2% contribution weight on average, with a median of 92.7%. For r/news we found the average to be 89.4%±9.4% with a median of 92.5%, which suggests that using hard assignments for our LDA topics here is appropriate.

**Figure 4:**
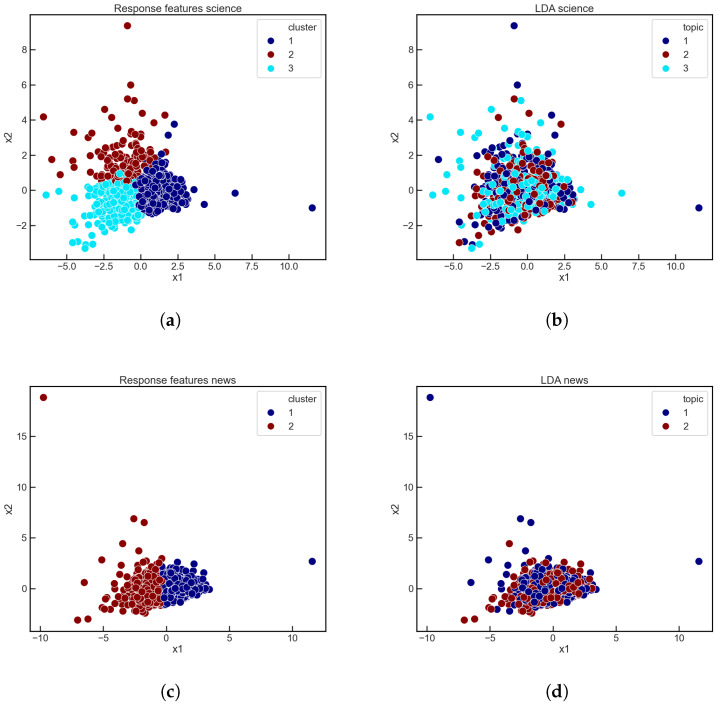
Comparison of clustering patterns with response feature K-means (**a**,**c**) and LDA (**b**,**d**) on the Subreddits r/science (**a**,**b**) and r/news (**c**,**d**).

We then color-coded each submission by their associated dominant topics and by their K-means cluster assignment, presenting these submissions projected into 2D space by PCA on their corresponding response features. We show a side-by-side comparisons of these two types of groupings in Figure 4. As seen in the figure, the distributions of topics do not match in any way, as the LDA topics do not group submissions in a similar fashion as the response features. While this does not indicate any kind of superiority of either model, the comparison here establishes that there is no correlation between such established NLP topic modeling processes and our novel approach to semantic grouping. Specifically, this comparison shows that the latent dynamics captured by our response features cannot directly be tied to textual analysis, and are distinctly independent from NLP, accentuating the text-agnostic component of this paradigm.

### 3.4. Outlier Analysis

**Figure 5:**
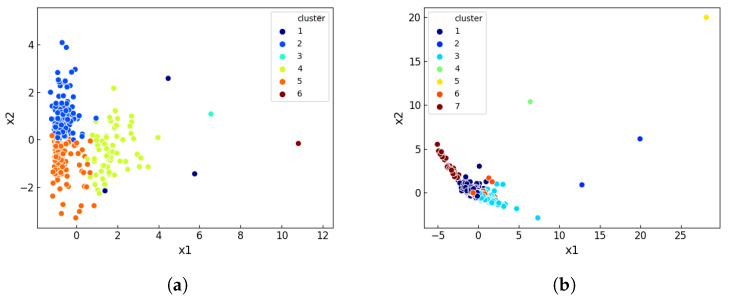
PCA of response features for 1000 submissions, clustered with K-means, from (**a**) r/soccer where K=6 and (**b**) r/gaming, where K=7.

In both examples of Figure 3, the overlap in top keywords decreases with the increase in Euclidean distance between the cluster centroids. This indicates a clear correlation between topical separation of submission content and the distance between submissions as represented through our response features. As such, an outlier should suggest a substantial difference in the content of the outlier submission and the rest of submissions being considered. Similar to the previous section, we consider 1000 submissions. We cluster them using K-means to establish different topical groupings, then isolate outliers using an Isolation Forest. For r/soccer, we set K=6. After clustering all the submissions, we plot them in 2D space using PCA and present them in Figure 5a.

Within Figure 5a, there are three clusters containing the majority of the submissions. In order to understand the reasons behind the differences in comment cascades between inliers and outliers, we manually investigate the title and comments of the involved submissions. However, before identifying our outlier, we first explore the textual differences in topic between the primary clusters. While $C_{4}$ contains only general discussions, $C_{2}$ and $C_{5}$ consist exclusively of “Match Thread” submissions. While in this example there is no clearly apparent textual disparity between these “Match Thread” clusters, we do discover that there is a more subtle degree of underlying differences. Supplemental analysis of the comments reveals that submissions in $C_{2}$ are often subject to more extensive discourse, as opposed to submissions in $C_{5}$, which tend to only have surface-level conversations. Thus, while there are few text-based dissimilarities between the two clusters, the response features are still able to identify additional latent differences in the similar topics visible only through user reactions.

When using our isolation forest on this data, we find that the rightmost point in $C_{6}$ has one of the highest anomaly scores and is one of the more distant points in Euclidean space with respect to the majority of other response features. Extracting the title and discussion from this outlier, we find this submission discusses a scandal involving FIFA concerning Qatar hosting the World Cup for soccer. Compared to the other submissions in our set, this is substantially different, inciting outrage from its repliers. However, it is important to note that this outlier, while different from its surrounding submissions, cannot be considered a “viral” post from just this classification as a response feature outlier. The virality of a post generally depends on the post receiving a large, disproportional number of responses, likes, or attention in a comparatively short amount of time. As this approach identifies response feature (and thus topical) abnormalities, some of the indicated submissions might be viral due to their significant topical differences (and likely novelty), but they are not necessarily guaranteed to be.

We compute another example of clustering and outlier detection in the r/gaming Subreddit. As before, we extract the response features of the collected 1000 submissions and cluster them using K-means, where K=7. The PCA of the response features is shown in Figure 5b. While a majority of submissions can be found in $C_{3}$ and $C_{7}$, there are a few outliers.

Like in our r/soccer example, when we analyze the text of the greatest group of submissions, we find that $C_{7}$ primarily contains video game giveaways, while $C_{3}$ consists of more general discussions. When using an isolation forest, we find that the singular point in $C_{5}$ is considered the greatest outlier of the submission set in terms of the anomaly score. Investigating this submission, we find that the involved text contains a story about a person who beat cancer and stayed emotionally strong, thanks to video games. In the context of r/gaming this can be seen as a very appealing story. Additionally, past work has confirmed that affect-laden text tends to drive the appeal (and often virality) of content [18], and the influx of supportive responses in this outlier confirms this. Our response features characterize this resultant cascade, identifying the significant difference in discourse dynamics such that this submission can be easily identified by an isolation forest.

## 4. Discussion

In this paper, we introduced a novel approach to analyzing social media information cascades using only the audience reactions triggered by a source post. Following this approach, we designed unique features to represent different aspects of discourse dynamics. Using these measures as response features to characterize an information cascade, we validated the effectiveness of these representative features through label classification and topic clustering, showing that response features can identify the differences between Subreddit genres without needing text. We then explored the relationship between cluster centroid distances of response features grouped through K-means, and the differences in keywords extracted from the clustered submission content. We found that the farther apart any two response feature clusters, the more topically distinct the involved submissions. This indicates that the dynamics of discourse and conversation that result from a submission (as characterized through our response features) can capture unique differences in topic both between genres and even more finely in a single genre itself. We then compared the submission clusters found with the response features against more traditional NLP topic modeling. Using a LDA model where the number of topics is equal to the number of response feature clusters, we investigated the correlation between LDA topic distribution and the semantic groupings discovered by the response features. We found that there was no correlation between the LDA topic distributions and the response features, indicating that the latent discourse patterns that the response features are using are distinctly independent of such NLP approaches. As an extension of our clustering analysis, we used outlier detection within particular Subreddits to identify topically aberrant submissions that stand out from the norm in terms of response features. We found that the identified outliers tend to involve unique, shocking, affect-laden, or generally novel content that is highly likely to spawn intense discussions and deep comment cascades that can be characterized by the response features in a way that sets it apart from the norm without ever using textual analysis for assistance and enhancement. Ultimately, we introduced a paradigm for identifying topics within social media using text-agnostic, reaction-based features that comes with multiple advantages, including the ability to identify latent semantic sub-divisions in a single topic, and the ability to isolate submissions that are topically aberrant from surrounding content without directly using text comparisons. In the future, we seek to integrate our response features into a pipeline that can analyze content generated from Reddit in real time, actively characterizing submissions and tracking outliers. Furthermore, while our paradigm for characterizing topics through reactions is text-agnostic, NLP and response features are not mutually exclusive. Therefore, we also plan to enhance our response features with text through multimodal embeddings, as text could improve topic classification (both across and within genres) and the detection of outliers.

## Figures and Tables

**Table 1:** Symbols and definitions.

Symbol	Definition
*S*	Some Subreddit within Reddit.
*M*	All users subscribed to *S*.
*r*	A submission within *S*, represented as a set of users that responded to the submission, r={r},r⊆M.
T(N,L)	A tree network representing the structure of hierarchically linked comments made by responders of a submission.
*n*	A user-generated comment within *T*, n∈N where *N* is the total set of comments within *T*.
$n_{0}$	The head node. This is the text submitted to the Subreddit that triggers the comment cascade.
*l*	A directed edge within *T*, representing the direction of information flowing between comments (from respondee to responder), l∈L where *L* is the total set of edges within *T*.
*B*	The set of branches found in *T*. $B_{i}=\left\{n\right\},B_{i}\subseteq N$ where the *i*th branch contains some subset of linked comments (including $n_{0}$) generated for a submission.

**Table 2:** Classifier scores for combinations of Subreddit labels.

**S**	politics, gaming, soccer	politics, gaming	politics, soccer	gaming, soccer	politics, atheism
**Score**	0.81	0.82	0.96	0.91	0.76

**Table 3:** Comparison of keyword frequency between the two identified topic clusters in r/hockey.

	Game Thread	Playoff	Series	Friday	Trash Talk
**Cluster 1 Frequency**	204	76	35	1	1
**Cluster 2 Frequency**	1	1	22	31	32

## Data Availability

Data, as found in [10], are available at http://goo.gl/sHUfhC, accessed on 27 March 2019. The code used for this analysis is available at https://github.com/Aganonce/TARGET, accessed on 7 July 2021.

## Supplementary Materials

The following are available online at https://www.mdpi.com/article/10.3390/e23121642/s1: Figure S1: Confusion matrices for the Support Vector Machine classifications of Subreddits, complementing Table 2 from the main text. The order of the figures corresponds to the order of results in the table. Figure S2: Distribution of comment response counts for all submissions. For each submission in the Reddit dataset, we computed the total number of comments (|N|), and binned all submissions by their corresponding counts. Note how the result follows the power law. Table S1: Classifier scores for combinations of Subreddit labels, grouped by the different comment ranges used to filter the submission pools.
