# Supplementary material for: Characterizing Topics in Social Media Using Dynamics of Conversation
Source: Entropy (Basel). 2021 Dec 7;23(12):1642. doi: 10.3390/e23121642 (PMC8700409; doi:10.3390/e23121642)
Supplement: Supplementary file 1 [file entropy-23-01642-s001.zip › entropy-1486783-supplementary.pdf]

Supplementary Materials for
Characterizing Topics in Social Media using Dynamics of Conversation

James Flamino, Bowen Gong, Frederick Buchanan, Boleslaw K. Szymanski

Supplementary Text

Testing for differences in response features across comment ranges

While in the main text we commonly test response features using pools of submissions that have a set comment range of $1000-3000$, it is important to investigate if these comment ranges can affect performance quality of the response features, and if the comment range established in the main text is a bound of predictive quality in our classification model. To do this, we repeat our genre classification test from the main text, extracting two sets of 1000 submissions from two different Subreddits, and training a SVM to differentiate between Subreddit. However, for this particular test we repeat this process three separate times for three different comment ranges: $100-500$, $500-1000$, and $1000-3000$. The last comment range is larger than the others to ensure that we can collect 1000 viable submissions for this pool, as the distribution of the total number of comments per submission follows a power law (see Figure S2). Given our 1000 submissions from each comment range, we consider three different combinations of Subreddits to train the SVM off of: politics and gaming, gaming and soccer, and politics and atheism. As before, we split the considered submission pool into training and testing data using a $70\%/30\%$ train/test split and extract their corresponding response features to use in the SVM. The results are shown in Table S1.

Note that the SVM scores for the three combinations of Subreddits within the $1000-3000$ comment range are different from the scores found in Table 2 in the main text. This is due to

		Comment Range		
		100 to 500	500 to 1000	1000 to 3000
**S**	Politics, gaming	0.81	0.85	0.83
	Gaming, soccer	0.81	0.91	0.96
	Politics, atheism	0.7	0.73	0.81

Table S1. Classifier scores for combinations of Subreddit labels, grouped by the different comment ranges used to filter the submission pools.

the fact that we randomly re-sampled our dataset for all the considered submission pool for this test. Regardless, the scores between the above table and Table 2 remain consistent. As seen in Table S1, the scores generally increase with the increasing number of comments allowed in the comment ranges. This is due to the fact that the more comments allowed within a submission, the more varied, multifaceted, and detailed the associated comment tree becomes. This allows for our response features to create a more detailed characterization of the submission through these responses, which in turn increases the quality of prediction of the SVM, as it now has high quality predictors from which to differentiate Subreddits.

Testing for differences in response features across years

For robustness, we check to see if there is any difference in response features within singular Subreddits between the first half of the Reddit dataset (2006 to 2010) and the second half (2011 to 2014). To do this, we consider the Subreddits politics, atheism, and science. For each Subreddit, we extract 1000 submissions for the first time range and 1000 submissions for the second time range, where all submissions were within the comment range of $100 - 3000$. The comment range here was expanded from the established range in the main text to ensure we could collect a sizeable amount of submissions from both time ranges. We extract the corresponding response features of each submission within the two groups and then assign ground truth labels of 0 and 1 to the submissions of each group, respectively. Joining these groups together we shuffle them and use a $70\%/30\%$ training/testing split to get our training and testing subsets. We train a Support Vector Machine Classifier (SVM) on the training subset and then test the SVM on the testing subset to see if there is any significant difference in response features from $2006 - 2010$ and $2011 - 2014$. For politics, the SVM yielded a score of 0.6. For atheism, 0.61. For science, 0.56. Since this is a binary classification, these results indicate that there is no significant difference in response features from $2006 - 2010$ and $2011 - 2014$. Intuitively this makes sense, as the lack in difference in response features across these two time ranges suggest that the conversational patterns that the response features are characterizing do not change with time very quickly.

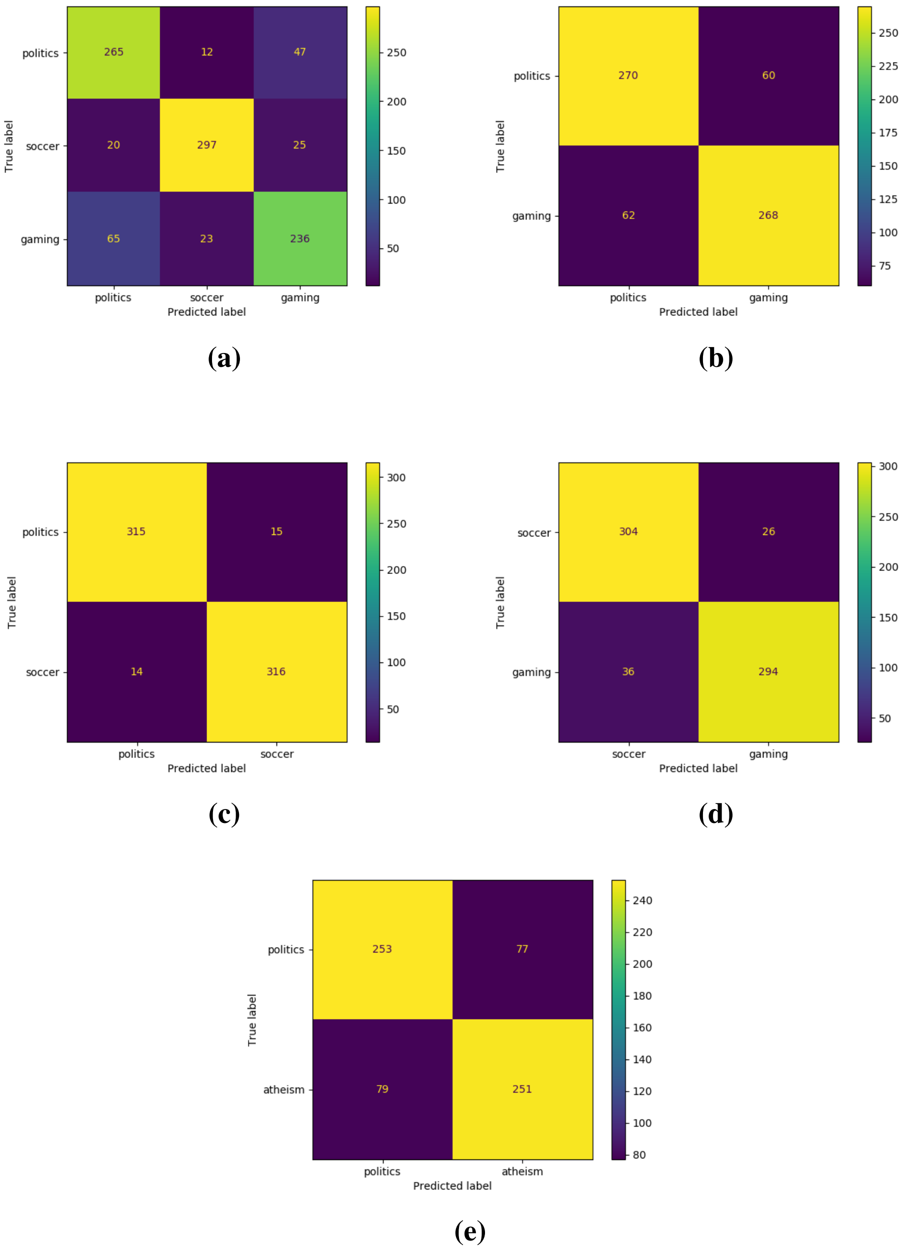

Figure S1. Confusion matrices for the Support Vector Machine classifications of Subreddits, complementing Table 2 from the main text. The order of the figures corresponds to the order of results in the table.

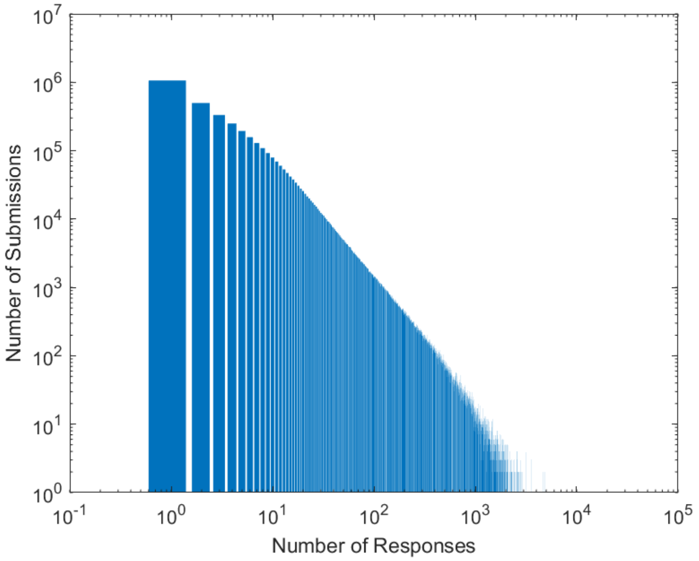

Figure S2. Distribution of comment response counts for all submissions. For each submission in the Reddit dataset, we computed the total number of comments ($|N|$), and binned all submissions by their corresponding counts. Note how the result follows the power law.
